# Enhancing Mental Health Research Publication from Low and Middle-Income Countries

**Published:** 2004

**Authors:** Shekhar Saxena, Pratap Sharan

**Affiliations:** 1 Co-ordinator, Mental Health: Evidence and Research; 2 Medical Officer, Mental Health: Evidence and Research, Department of Mental Health and Substance Abuse, WHO, Geneva, Switzerland


*Indian Journal of Psychiatry, 2004, 46(I)72-78*

### Enhancing Mental Health Research Publication from Low and Middle-income Countries

**Shekhar Saxena*[1] Pratap Sharan[2]**

Recent epidemiological research has demonstrated that mental disorders cause considerable burden on individuals, communities and health services and it is projected that the burden will increase in the coming years (Ustun and Sartorius, 1995; Murray and Lopez, 1997; Ayuso-Mateos, 2003; WHO, 2003). Preliminary studies from India confirm these findings (Velayudhan et al, 1998; Girish et al, 1999; Chisholm et al, 2000; Pal et al, 2000). In contrast, the resources available to meet mental health challenges in low- and middle-income (LAMI) countries is meager - an overwhelming majority of countries in African and South East Asia regions including India spend less than 1% of their limited health budgets on mental health (WHO, 2001 a, 2001 b, 2001 c). As a result, mental health services are poorly developed in these regions, e.g. India has 0.25 beds per 10,000 population, of which only 20% are in the community setting. It has 0.4 psychiatrists per 10,000 population and inexplicably only one-tenth as many psychiatric nurses. Hopefully, things will look up following the recent Supreme Court directives and Government of India action. Research shows that in high-income countries, between 44% and 70% of patients with common and severe mental disorders do not receive any treatment (Kohn et al, 2004). The treatment gap in developing countries could be closer to 90%. Closing this gap is a clear obligation; otherwise no discourse around new classifications, concern about more sophisticated diagnosis, or development of innovative psychopharmacological research can be credible (Saraceno, 2004).

### Role of Mental Health Research

The Mental Health: Global Action Programme (mhGAP) of WHO envisions an active role for research in the multidimensional efforts required to change the current mental health situation at country level (WHO, 2002). Research-generated information is perceived as essential to determine needs; to propose new cost-effective interventions of an individual or collective nature, to monitor the process of their implementation and evaluate the changes sought; and to explore the obstacles that prevent recommended cost-effective action to be carried out (WHO, 2001 a). Research-generated information should enable LAMI countries to better utilize their resources.

Currently, the mental health effort in LAMI countries is based primarily on evidence from high-income countries, which have vastly different cultural and socio-economic contexts. However, culturally relevant research is crucial for mental health policy and service development, treatment decision-making, and anti-stigma and discrimination programmes. Similarly, mental health research in relation to LAMI countries that is done by academics from high-income countries often has no real connection to local service development. Such research forms 22.1% of the mental health literature available on India (Saxena et al, 2004).

### Strategies to Advance Research in LAMI Countries

First, it is necessary to set a priority agenda, viable and zealously tailored to country mental health needs, characteristics and resources (Saxena et al, 2004). To be sustainable, the research policy for a country should be developed in harmony with various components of the national mental health policy, and with other health and developmental priorities (Mari et al, 1997; Patel, 2000). The Indian Psychiatric Society (IPS) and Indian Council of Medical Research (ICMR) along with other professional bodies can play a major role in placing mental health research on the agenda of policy makers and planners in India. They are also well placed to carry out credible advocacy that is needed to convince donors of the significant public health burden of mental disorders in India. Second, a research-friendly cultural environment needs to be created by involving policy makers, programme planners and managers, governmental officials, mental health advocates, professionals, users and carers, and university settings of LAMI countries. Third, a major undertaking on capacity building and infrastructural support is required. Probably the best ways of doing this is to promote (truly) collaborative research and provide technical/scientific support to institutions in LAMI countries. The IPS, ICMR and other professional bodies can play a key role in collaboration with

[1]Co-ordinator, Mental Health: Evidence and Research

[2]Medical Officer, Mental Health: Evidence and Research, Department of Mental Health and Substance Abuse, WHO, Geneva

*Correspondence: Shekhar Saxena, Coordinator, Mental Health: Evidence and Research, Department of Mental Health and Substance Abuse, World Health Organization, Geneva 27, CH 1211, Switzerland Email: saxenas@who.int Tel : +41 22 7913625, Fax: + 41 22 7914160

academic institutions to strengthen research training programs in the country. Fourth, methodologies that do not require sophisticated infrastructural support should be made available widely. Finally, a systematic approach including attention to the relevance of research questions, early involvement of practitioners, policy makers, advocacy groups and family members/consumers, and judicious use of media should be formulated in order to enhance the utilization of research for policy and practice.

The research efforts from LAMI countries will have greater chances of success if research institutions in high-income countries (in India, fortunately, prestigious research institutions within the country can play this role), research foundations and country donors, and editors of scientific journals provide their fullest and sustained support to national and regional efforts in LAMI countries.

### Role of Scientific Journals Publishing Mental Health Research

Public mental health in LAMI countries can be improved by facilitating the generation and flow of information. Scientific journals can play a major role in achieving these ends. However, current publication environment is not facilitative of this. Authors have reported that LAMI countries contribute approximately 6% of articles to leading psychiatry journals (Patel and Sumathipala, 2001; Parker and Parker, 2002). Of these approximately one-tenth relates to India (Saxena et al, 2004). In terms of proportions it amounts to 0.6% of world mental health publications for approximately 17% of world population. Even more worrying is the fact that in Biomedical publications, the gap between countries with low and high level of publications is widening (Perez-Iratxeta and Andrade, 2002). While, it is true that less mental health research is done in LAMI countries due to low priority for such research, lesser resources, limitations in research capacity and difficulties in reporting research due to language barriers, a reduced appreciation of the research needs of LAMI countries at the level of reviewers and editorial boards of international journals could also play a part. Only one out of a total of 530 editorial and advisory board members of ten psychiatric journals with the highest impact factor rating for the year 2000 was based in India (Saxena et al, 2003). Also, there is a tendency among Biomedical journals to send manuscripts to reviewers within their own region (Garrow et al, 1998). The absence of well-informed interlocutors familiar with research needs of LAMI countries could lead to a bias against publication of research from or about these countries.

Journals in LAMI countries, besides being a repository of local mental health wisdom, can also help in educating their authors and in translating mental health research into action through dissemination of relevant local and international information to policy makers and public health officials in their countries and regions. However, most mental health journals based in LAMI countries face a multitude of problems including those of resources for publication and dissemination (financial, managerial, marketing), editorial skills and review process, author pool and language, and perhaps biases in indexing systems (Gibbs, 1995). An overwhelming majority (98%) of biomedical journals indexed in international databases are from the developed world (Edejer, 2000). It is a sad fact that after more than 50 years of regular publication, the Indian Journal of Psychiatry is not indexed in the Index Medicus or the science citation index of the Institute for Scientific Information (ISI). The lack of visibility of journals from LAMI countries affects their dissemination opportunities (e.g. libraries in LAMI countries also subscribe mainly to influential journals from Western countries [Cetto and Alonso-Gamboa, 1998]), and consequently the possibility of application of research results carried by them.

LAMI countries have very limited access to scientific materials because of the expenses involved. The crisis is worsening as in the last couple of decades, the subscription costs of many scholarly journals (especially those published by powerful commercial publishers) have escalated at a rate far exceeding the rate of inflation (Horton, 2003). In addition, many new journals have been started. Even large academic libraries in high-income countries have had to be fairly selective about subscribing to journals and have indeed carried out extensive journal-cancellation projects in the last few years. Some improvement has occurred in electronic dissemination of research, due to the cost-effectiveness of this medium. A number of initiatives (e.g. WHO sponsored Health Internet work Access to Research Information [HINARI], Scientific Electronic Library Online [SciELO] project of Latin America), attempt to provide access to electronic literature, in addition to similar attempts by scholarly societies and journals (Momen, 2003). Open access journals aim to free publishing from copyright controls besides providing free access to the contents of their journals. These initiatives are beginning to have an impact, but a lot still needs to be done, since freely accessible literature forms only a small proportion of scholarly publishing and LAMI countries have limited access to internet itself. Similarly, levying of page charges makes it difficult for authors from LAMI countries to publish in open access journals unless page charges are subsided by research funding agencies or governments (Horton, 2003; Momen, 2003).

### Editors pledge to support mental health research efforts from LAMI countries

A well-managed program for supporting mental health research publication is required to address the needs of researchers and journals from LAMI countries. The Department of Mental Health and Substance Abuse, World Health Organization and the Bulletin of the World Health Organization, organized an international meeting on 'Mental Health Research in Developing Countries: Role of Scientific Journals' on 20 and 21 November, 2003 in Geneva, Switzerland. It was attended by twenty-five editors representing mental health journals and general and public health journals publishing mental health research including the Indian Journal of Psychiatry and the National Medical Journal of India. A number of other editors reviewed and contributed to the background and follow-up material.

Issues related to 'Responsibility of scientific journals towards international mental health,' 'Supporting mental health researchers from LAMI countries,' 'Supporting mental health journals from LAMI countries,' and 'Enhancing dissemination of mental health research publications' were discussed. A joint statement (Appendix I) was issued by the participants (Appendix II). A catalogue of ideas (Appendix III) was also developed to guide follow-up actions by individual journals and editorial and international organizations.

### Role of LAMI country journals publishing mental health research

There is much that journals in LAMI countries can do to help themselves and each other. They can participate more actively in international communication on mental health (e.g. by inviting researchers from other countries to serve on review and editorial panels, developing twinning or pairing arrangements with established journals). They can strive to develop author (e.g. by offering support in preparation of articles, organizing workshops for researchers) and editorial (e.g. organizing workshops for reviewers and editors) capacity in the region. They could also focus on dissemination (e.g. online publication, publication of abstracts/summaries/articles in more than one language) and utilization of mental health research outputs towards improvement of mental health services and quality of care in the region (e.g. dissemination of information with public health relevance to policy makers and general media). They can only gain from these efforts as improvement in the standard of the journal often has the spin-off of attracting more quality articles and financial (and other) resources for the journal.

### REFERENCES

Ayuso-Mateos, J.L. (2003). The global burden of mental and neurological disorders. Journal of Mental Health Policy and Economics, 6 (Supplement 1), S4.

Cetto, A.M., Alonso-Gamboa, O. (1998). Scientific periodicals in Latin America and the Caribbean: a global perspective. Interciencia, 23, 84-93.

Chisholm, D., Sekar, K., Kishore Kumar, K., Saeed, K., James, S., Mubbashar, M., Murthy, R.S. (2000). Integration of mental health care into primary care. Demonstration cost-outcome study in India and Pakistan. British Journal of Psychiatry, 176, 581-588.

Edejer, T.T. (2000). Disseminating health information in developing countries: the role of the internet. BMJ, 321, 797-800.

Garrow, J., Butterfield, M., Marshall, J., Williamson, A. (1998). The reported training and experience of editors in chief of specialist clinical medical journals. Journal of the American Medical Association, 280, 286-287.

Gibbs, W.W. (1995). Lost science in the Third World. Scientific American, 273, 92-99.

Girish, K., Murthy, P., Issac, M.K. (1999). Drug treatment in schizophrenia: issues of comparability and costs. Indian Journal of Psychiatry, 41, 100-103.

Horton, R. (2003). 21st-century biomedical journals: failures and futures. Lancet, 362, 1510-1512.

Kohn, R., Saxena, S., Levav, I., Saraceno, B. (2004). Treatment gap in mental health care. Bulletin of the World Health Organization (In Press).

Mari, J., Lozano, J.M., Duley, L. (1997). Erasing the global divide in health research. BMJ, 314, 390.

Momen, H. (2003). Equitable access to scientific and technical information for health. Bulletin of the World Health Organization, 81, 700.

Murray, C.J.L. & Lopez, D. (1997). Alternative projections of mortality and disability by cause 1990-2020: global burden of disease study. Lancet, 349, 1498-1504.

Pal, H.R., Saxena, S., Chandrashekhar, K., Sudha, S.J., Murthy, R.S., Thara, R., Srinivasan, T.N., Gupta, D., Singh, U. (2000). Issues related to disability in India: a focus group study. National Medical Journal of India, 13, 237-241.

Parker, G. & Parker, K. (2002). A profile of regional psychiatry publishing: home and away. Australian and New Zealand Journal of Psychiatry, 36, 693-696.

Patel, V. (2000). The need for treatment evidence for common mental disorders in developing countries. Psychological Medicine, 30, 743-746.

Patel, V. & Sumathipala, A. (2001). International representation in psychiatric literature. British Journal of Psychiatry, 178, 406-409.

Perez-Iratxeta, C. & Andrade, M.A. (2002). Worldwide scientific publishing activity. Science, 297, 519.

Saraceno, B. (2004). Mental health: scarce resources need new paradigms. World Psychiatry, 3, 3-5.

Saxena, S., Maulik, P., Levav, I., Saraceno, B. (2003). How international are the editorial boards of leading psychiatry journals? Lancet, 361, 609.

Saxena, S., Saraceno, B., Levav, I. (2004). Research for Change. World Health Organization, Geneva (In Press).

Ustün, T.B. & Sartorius, N. (1995). Mental Illness in General Health Care: An International Study. John Wiley & Sons, Chichester.

Velayudhan, A., Benegal, V., Jain, S. (1998). Social cost of alcoholism. Indian Journal of Psychiatry, 40, 1-2.

World Health Organization. (2001 a). The World Health Report 2001. Mental Health. New Understanding, New Hope. World Health Organization, Geneva.

World Health Organization. (2001 b). Atlas: Mental Health Resources in the World 2001. World Health Organization (WHO/NMH/MSD/MDP/01.1), Geneva.

World Health Organization. (2001 c). Atlas: Country Profiles on Mental Health Resources 2001. World Health Organization (WHO/NMH/MSD/MDP/01.3), Geneva.

World Health Organization. (2002). Mental Health Global Action Programme. World Health Organization, Geneva.

World Health Organization. (2003). The World Health Report 2003. Shaping the Future. World Health Organization, Geneva.

### Appendix I: The Joint Statement

### Galvanising Mental Health Research in Low- and Middle-Income Countries: Role of Scientific Journals

The Department of Mental Health and Substance Abuse, WHO organized a meeting on ***Mental Health Research in Developing Countries: Role of Scientific Journals*** in Geneva on 20 and 21 November 2003 that was attended by twenty-five editors representing journals publishing mental health research. A number of other editors reviewed and contributed to the background and follow-up material. This statement is issued by all participants jointly.

Research is needed to address the enormous unmet mental health needs of low- and middle-income (LAMI) countries. Scientific journals play an important role in production and dissemination of research. However, at present, only a minute proportion of research published in widely accessible mental health and psychiatric journals is from or about these countries. Yet over 85% of the world's population lives in the 153 countries categorized as low- and middle-income, according to World Bank criteria. Even more worrying is the observation that the gap between these and high-income countries may be widening in terms of their number of publications. The meeting was aimed at finding ways of resolving this unsatisfactory situation.

### Responsibility of scientific journals towards international mental health

Science, in its quest to accomplish valid generalisations about nature, is inherently global. Researchers from all parts of the world should, desirably, contribute to new knowledge about mental health and mental illness, and publish their reports in widely accessible journals. This process is facilitated by a shared understanding of aims and scientific methods, formats of presentation and reference to previous published work. Mental health research from LAMI countries is needed for advocacy, policy development, establishment and expansion of clinical services and to educate investigators in research skills. A steady stream of information about mental health issues in these countries would also contribute to a greater international and multicultural understanding of mental health and ill-health.

Unfortunately, substantial barriers impede publication of mental health research from LAMI countries in widely accessible journals. Researchers from LAMI countries are often unable to meet the requirements of these journals because of limited access to information, lack of advice on research design and statistics, difficulty in writing in a foreign language, and overall material, financial, policy and infrastructural constraints. Limited appreciation of the research needs of, and realities in LAMI countries and the comparative anonymity of their researchers and research centres in editorial offices of journals may constitute additional barriers. Many researchers from LAMI countries are daunted by the seemingly insurmountable chasm between their research effort and its publication in international journals.

### Supporting mental health researchers from low- and middle-income countries

We need to face the challenge of reducing the barriers to publication of mental health research by investigators working in LAMI countries. Time, skills, resources and commitment are needed to publish relevant studies from these countries. 'Editors and reviewers' experience with and interest in LAMI countries could be an asset in facilitating publication. Meeting researchers from these countries on 'their home ground' could assist this process. International journals could also help researchers improve their submissions by diligent assessment, detailed recommendations for revision and sympathetic consideration of revised versions, even if it means requesting reviewers to 'take an extra round' to make papers suitable for publication. This is not to say that journals need to lower their standards in publishing papers from LAMI countries; rather, they should devise strategies to help authors attain those standards. Other approaches to support contributions from LAMI countries could be to launch 'starter' sections such as information pages and special columns or even dedicated issues of the journal.

Capacity building is the paramount factor in the long term. Training in research methodology and scientific writing is needed. This could be done through mentoring, personal encouragement, training courses and research collaboration. Increased access to mental health research publications would, by itself, help in capacity building.

### Supporting mental health journals from low- and middle-income countries

A major impediment in accessing mental health research from LAMI countries is the lack of visibility of journals published in these countries. Most of them are not indexed in international databases and are often not available beyond their country or region of origin. These journals are published under strained circumstances, in that they often lack sound financial support and have a hard time becoming self-sufficient. They also have difficulty in obtaining suitable articles for publication because their author pool is limited; moreover, influential authors from this pool prefer to publish their best research in indexed journals. Some authors who submit their articles to LAMI country based journals may have limited skills in conducting research and/or in writing up their reports. However, it must be stressed that some excellent work does find publication in these journals.

The task of strengthening journals in LAMI countries begins from the recognition of their role as contributors to the enhancement of the mental health knowledge base and as partners in the international research community. Editors of LAMI country based journals require support to elevate standards in editorial procedures, peer review and overall journal management since sufficient expertise and experience may be lacking. This could be achieved through their participation in the publication process of established journals, mentorship, twinning arrangements and training workshops.

**Enhancing dissemination of mental health research publications**

Many high quality mental health journals have a wide distribution, but most of their subscribers are from high-income countries. Special attention to dissemination of research findings is needed urgently in order to maximize their impact on mental health policy and practice and advance relevant research in LAMI countries. Increasing online availability is cost-effective since little additional expenditure is required to provide access to new users apart from the initial costs of posting material on a website. Free access to many categories of electronic resources is provided by many journals. Initiatives such as the WHO-led Health Inter Network Access to Research Initiative (HINARI) offer institutions in LAMI countries electronic access to thousands of journals at no or very low cost. The Open Access model provides free online access along with the possibility of unrestricted dissemination of research materials, but charges for publication may be prohibitive for authors from LAMI countries unless support comes from funding agencies and governments, e.g. the Scientific Electronic Library Online (SciELO) project in Latin America. Governments in other LAMI countries need to be made aware of the opportunities provided by information technology for dissemination and application of research knowledge.

**The role of various stakeholders**

Editors of journals, editors' associations and international organizations, including WHO could help achieve the aforementioned objectives. A catalogue of ideas is presented to act as a starting point for specific action. Although these ideas have been developed for the field of mental health, many of them may apply to other areas of health.

**Appendix II: Signatories to the Joint Statement**

***Acta Psychiatrica Scandinavica*** (Povl Munk-Jorgensen), ***American Journal of Orthopsychiatry*** (Carlos Sluzki), ***Annals of General Hospital Psychiatry*** (George St. Kaprinis, Konstantinos N. Fountoulakis), ***Anthropology and Medicine*** (Sushrut Jadhav), ***Australian and New Zealand Journal of Psychiatry*** (Sidney Bloch), ***BioMed Central Psychiatry*** (Pritpal S. Tamber), ***British Journal of Psychiatry*** (Peter Tyrer), ***BMJ*** (Kamran Abbasi), ***Bulletin of World Health Organization*** (Hooman Momen), ***Child Abuse and Neglect, The International Journal*** (John M. Leventhal), ***Chinese Journal of Nervous and Mental Disease*** (Li Yingxi, Guan Jinli), ***Comprehensive Psychiatry*** (David L. Dunner), ***Culture, Medicine and Psychiatry*** (Mary-Jo Delvecchio Good), ***Epidemiologia e Psichiatria Sociale*** (Michele Tansella), ***L'Evolution Psychiatrique*** (Yves Thoret), ***Indian Journal of Psychiatry*** (Utpal Goswami), ***L'Information Psychiatrique*** (Thierry Tremine), ***International Journal of Social Psychiatry*** (Dinesh Bhugra), ***International Psychiatry*** (Hamid Ghodse), ***Journal of Child and Adolescent Mental Health*** (Alan Flisher), ***Journal of Nervous and Mental Disease*** (Eugene B. Brody, Kathy McKnight), ***Lancet*** (Laragh Gollogly), ***Primary Care Psychiatry*** (Sean Lynch), ***Psychiatry: Interpersonal and Biological Processes*** (Robert Ursano), ***Psychiatry Research*** (Monte Buchsbaum), ***Psychological Medicine*** (Eugene Paykel), ***Psychology and Psychotherapy: Theory, Research and Practice*** (Phil Richardson), ***Psychopathologie Africaine*** (Momar Gueye), ***Quarterly Journal of Pakistan Psychiatric Society*** (Amin A. Gadit), ***Revista Brasileria de Psiquiatria*** (Jair Mari), ***Salud Mental*** (Hector Perez-Rincon), ***Social Psychiatry and Psychiatric Epidemiology*** (Paul Bebbington), ***South African Journal of Psychiatry*** (Robin Emsley, Susan Hawkridge), ***Transcultural Psychiatry*** (Laurence J. Kirmayer), ***World Psychiatry*** (Mario Maj), ***Forum for African Medical Editors*** (James K. Tumwine), ***Global Forum for Health Research*** (Andres de Francisco), ***World Association of Medical Editors*** (Ana Marusic, Peush Sahni), ***World Health Organization*** (Shekhar Saxena, Pratap Sharan, Benedetto Saraceno, Barbara Aronson, Vladimir Poznyak, Izthak Levav, Edith Certain, R Srinivasa Murthy, Tikki Pang).

Shekhar Saxena, Pratap Sharan, Hooman Momen and Benedetto Saraceno organized the WHO Meeting leading to this joint statement.

**Appendix III: Catalogue of ideas**

**INDIVIDUAL JOURNALS**

**Giving priority to relevant mental health research from low- and middle-income countries**

- Educate editors and reviewers on research needs of and research infrastructure in LAMI countries;
- Use surveys of various stakeholders such as readers (including those from other regions) for shaping journals' priorities;
- Sensitize readers and other stakeholders to international mental health issues (e.g. through special sections and dedicated issues, guest editorship and the commissioning of relevant research from LAMI countries);
- Critically re-examine the use and limitation of measures such as citation rates and impact factors;
- Adopt a multilingual approach, such as translation of relevant articles and abstracts into other languages;
- Include reviewers and correspondents with a special interest and expertise in LAMI countries on editorial boards;
- Accept a higher proportion of submissions from LAMI countries for review; and
- Encourage general medical journals to publish mental health research especially in countries/regions where no mental health journal exists at present.

**Supporting authors/researchers from low- and middle-income countries**

- Familiarize researchers from LAMI countries with the peer review process;
- Provide constructive critical feedback/detailed recommendations for revision;
- Make provision for extra rounds of editing, assistance with language and use of technical editors;
- Pay attention to the educational goals of the review process (e.g. availability of reviewer's comments to readers or recruiting young researchers in LAMI countries to referee papers);
- Provide mentorship and support prior to submission;
- Organise training workshops for LAMI country researchers and students on scientific writing and research methodology;
- Facilitate the involvement of researchers in multi-centre projects and research groups;
- Accept and process submissions online; and
- Devise strategies to prevent economic exclusion of researchers from LAMI countries in author/input paying publishing models.

**Supporting journals from low- and middle-income countries**

- Support "twinning" or "pairing" arrangements, such as invited editorials, exchange of journals, cross-publication of contents/abstracts/summaries/articles and joint publications;
- Agree to serve on editorial boards or as reviewers;
- Agree to mentor reviewers and editors;
- Provide training workshops for editors and reviewers; and
- Support national/regional journals in developing their own websites and/or seeking inclusion in specialized websites on mental health

**Enhancing Dissemination**

- Participate in electronic dissemination initiatives or provision of free/open access through the journal's website;
- Participate in "buddy system"/peer sponsoring initiatives;
- Employ user-friendly technology for easier downloads;
- Subsidize journal subscriptions for LAMI countries; and
- Explore mechanisms for publication of selected papers in more than one journal for wider dissemination.

**EDITORS' ASSOCIATIONS**

- Develop guidelines for good editorial practice concerning publishing and research ethics and conflicts of interest;
- Facilitate access to literature and bibliographic services (e.g. through a directory of databases);
- Support authors to access appropriate specialized journals and specific audience (e.g. through a database of journals and instructions to authors);
- Facilitate mentoring for editors, reviewers and researchers;
- Organise training of editors, reviewers and researchers from LAMI countries; and

- Facilitate the multidirectional flow of articles, resources and expertise (e.g. translation of relevant articles and support with information technology).

### INTERNATIONAL ORGANIZATIONS

### Supporting mental health research, research infrastructure and publications

- Influence other international institutions to give priority to mental health research in their agendas for LAMI countries;
- Support national institutions in LAMI countries to urge their governments to give higher priority to mental health research;
- Support inclusion of researchers/editors from LAMI countries in relevant decision-making forums; and
- Facilitate capacity building for researchers and journals from LAMI countries.

### Enhancing Dissemination

- Assess information needs in LAMI countries and raise awareness of these;
- Provide access to journals publishing mental health research (e.g. expansion of HINARI or enabling journals to be open access); and
- Encourage and facilitate the application of information technology.

### Enhancing Collaboration

- Develop networks between editors, editorial organizations, professional bodies, publishers, funding agencies, national and international organizations and the media; and
- Adopt a systematic approach for follow up: statement of changes hoped for, development of outcome criteria, assessment of progress.

